# The Children, Caregivers, and Community (C3) study of together growing strong: A protocol for an observational, place-based initiative in Sunset Park, Brooklyn

**DOI:** 10.1371/journal.pone.0290985

**Published:** 2023-09-01

**Authors:** Elizabeth B. Miller, Caitlin F. Canfield, R. Gabriela Barajas-Gonzalez, Alicia Chung, Julie Katter, Bonnie D. Kerker

**Affiliations:** 1 Department of Population Health, NYU Grossman School of Medicine, New York, NY, United States of America; 2 Department of Pediatrics, NYU Grossman School of Medicine, New York, NY, United States of America; Federation University Australia, AUSTRALIA

## Abstract

Reaching population-level impact for families in poverty requires moving beyond a sole focus on individuals, to a wider focus on interactions between individuals and their broader environmental contexts. Place-based initiatives have emerged as a policy response to promote community-level change around these broader interactions between individuals and their local communities through addressing long-standing disparities in housing, employment, education, and health. Together Growing Strong (TGS) is one such place-based initiative focused on transforming the health, wellbeing, and development of young children and their families in Sunset Park, Brooklyn. The Children, Caregivers, and Community (C3) Study is an outcomes-based study designed to assess the trajectories of children and families in Sunset Park along indicators such as family health and wellbeing and child development in relation to TGS program participation. The aims, scope, and protocol of the C3 Study are the subjects of this paper.

## Introduction

Poverty-related disparities in child development and school readiness are observable very early in a child’s life, increase over time, and persist across the early grades of school without intervention [1]. Based on the stressors and constraints on families living in poverty, young children growing up in households with low incomes may have fewer opportunities for home-based learning, including limited access to print materials, lower levels of language exposure and complexity, and shared reading [2,3]. These disparities in early learning and language experiences contribute to variations in school readiness skills [4], which have been linked with later academic achievement and economic success over the lifespan [5,6].

Due to a history of racism in the United States (U.S.) that privileges white, English-speaking populations, children of color are more likely to live in poverty [7,8]. As such, children of color are often described as not meeting school-readiness milestones [9–11]. While this may, in part, be due to the correlation between race and poverty in the U.S., it may also be due to the use of assessments that use white culture as the norm and judge children’s progress in conforming to those norms [12,13]. This may be particularly problematic among children of immigrants, or immigrants themselves, as these measures often do not capture the cultural and linguistic assets that such children bring to the classroom [14].

Additionally, the neighborhood built environment that poor and non-white families often live in may reflect socio-political factors that historically disinvest in resources that affect access to air quality, green space, food, social services and housing–key social determinants that affect child development outcomes [15–19]. Analysis of the 2020–2021 National Survey of Children’s Health, for example, indicates that most Black children in the U.S. live in neighborhoods that lack amenities such as sidewalks, parks/playgrounds, libraries, and recreational centers [20]. Across the 100 largest metros in the U.S., Black children are 7.6 times and Hispanic children 5.3 times more likely to live in very low-opportunity neighborhoods than white children [21].

Living in disinvested neighborhoods and low-opportunity settings can have a negative impact on a child’s mental health outlook and other negative attributes that may affect their emotional, behavioral, and physical health, as well as academic achievement [22–25]. Early childhood adversity, including poverty, racism and discrimination, may also affect brain development and key child development outcomes that may have longitudinal effects later in life [8,26]. As a result, children of color living in historically disinvested neighborhoods face greater odds of these poor health outcomes, especially as they prepare for kindergarten [27].

Ecological [28] and life course [29] perspectives argue that human development is shaped by a nested series of interconnected systems that start at the individual child level and radiate outward to include community and wider culture. As such, research in early childhood highlights the importance of early environments and interactions with trusted caregivers for the development of early cognitive, language and literacy, and socioemotional skills [30–32]. Early experiences in the home, community, and systems that serve families represent an important modifiable factor in mitigating economic disparities in children’s school readiness [1,33]. Thus, achieving population-level change for families in poverty requires moving beyond a sole focus on individuals, to a wider focus on interactions between individuals and their broader environmental contexts, including social conditions, community-level factors, and social service, healthcare and education systems [34].

Place-based initiatives have emerged as a policy response to promote community-level change around these broader interactions between individuals and their local communities [35]. Such initiatives first emerged in the early 20^th^ century from the settlement house movement [36] and since the 1960s largely morphed into philanthropic and government efforts centered around places where families with low incomes are residentially concentrated to improve quality of life and economic opportunities [37]. Place-based initiatives have historically aimed to invigorate urban renewal by addressing long-standing disparities in housing, employment, education, and health caused by public policy decisions, market failures, and patterns of discrimination [38].

In 2017, a private family foundation made a generous financial commitment to NYU Langone Health to develop, implement, and evaluate a sustainable and replicable community-based approach to improve school readiness at kindergarten entry in Sunset Park, Brooklyn. From these funds, Together Growing Strong (TGS) emerged as a place-based initiative focused on transforming the health, wellbeing, and developmental trajectories of young children and their families in the community. Almost half (46%) of the population in Sunset Park is born outside the U.S. (35% identifies as Asian and 36% as Hispanic) [39]. It is a unique neighborhood in that it has both many assets and vulnerabilities. Families who live in Sunset Park have strong community ties with robust social networks, many community- and faith-based organizations, and committed community members.

Yet, families in Sunset Park also face high levels of poverty, low educational attainment, and high rent burden. Data from the American Community Survey 2017–2021 reported that 21% of Sunset Park residents reported living below the poverty level compared with 17% in New York City. Educational attainment was also lower, with 38% of Sunset Park residents reporting less than a high school degree compared with 17% across New York City. And, 30% of renter households in Sunset Park were severely rent burdened (i.e., spent more than 50% of household income on rent) [39]. Disparities in crowded housing conditions were also more prevalent in Sunset Park compared with the rest of the city; 21% of housing units in Sunset Park are crowded (1.01 or more occupants per room) compared with only 9% across the city [40].

In 2021, in line with the TGS goal of evaluation, the private family foundation made an additional commitment to fund an outcomes-based study, known as the Children, Caregivers, and Community (C3) Study, that would assess the trajectories of children and families in Sunset Park along short-term (family health and wellbeing) and long-term (child development) indicators in relation to TGS program participation. The aims, scope, and protocol of the C3 Study are the subjects of this paper.

## Methods

### Study design and setting

TGS is a place-based, community-centered initiative that seeks to integrate new and existing evidence-based programs (EBPs) in the community and strengthen systems serving families from the prenatal period through school entry. The initiative is grounded in an anti-racist and community centered perspective, with the ultimate goal of improving children’s school readiness across the community. As such, TGS conducts much formative research and shares data back with community members and other community stakeholders to interpret the data and plan/modify different components of the initiative. These components include individual-level EBPs (focused on child social-emotional health, early literacy and language skills, early relational health, and maternal social-emotional health/wellness); digital messaging services; workshops with community members; support for healthcare providers and early childhood educators; education, healthcare and social service systems-level work; and policy advocacy.

The C3 Study seeks to examine the trajectories of families in Sunset Park, Brooklyn, to better understand the potential impact of TGS. In particular, by observing families over time, the current study seeks to evaluate baseline assets and vulnerabilities for families across the Sunset Park community, as well as associations between TGS participation and short- and long-term indicators of family health and wellbeing and child development. As a result, this study will highlight family and community assets that allow children to thrive, as well as modifiable family- and community-level risk factors.

In order to examine these trajectories, we are conducting two separate studies of 1,350 families living in or receiving services in Sunset Park, Brooklyn: 1) a longitudinal observational cohort study with consecutive enrollment of 900 pregnant women over 3 years (*n* = 300/year) with follow-up of 1–2 assessments per year through age 6 as well as a qualitative component; and 2) a cross-sectional study of 450 parent-child dyads at age 2 years (*n* = 150), 4 years (*n* = 150), and 6 years (*n* = 150) for a one-time assessment, which will provide an indication of baseline functioning and serve as a historical control for the longitudinal study. Institutional Review Board (IRB) approval for the C3 Study was obtained from NYU Grossman School of Medicine (NYUGSOM; S22-00242), and the study has been preregistered at AsPredicted.org.

Data collection for the C3 Study includes structured parent interviews, observations of parent-child interactions, and direct child assessments. Assessments for both the longitudinal and cross-sectional studies last 1–2 hours each. Qualitative interviews will be conducted with a subset of the longitudinal sample, which are expected to last an additional 1–2 hours each. Informed consent is obtained from all study participants. Participants are compensated for their time and travel. This incentive payment is provided upon the completion of each study visit, and receipt of the payment is not contingent on successful completion of the study.

### Sample

#### Longitudinal study

Pregnant women who speak English, Spanish, or Chinese are currently being recruited from NYU-affiliated prenatal clinics in the Sunset Park neighborhood, including both private OB/GYN practices that are affiliated with NYU Langone faculty group practices and the Family Health Centers at NYU Langone which are federally-qualified health centers (FQHCs) that care for primarily Medicaid-eligible patients or those with low incomes. The patient populations at the NYU Langone faculty group practices and at the Family Health Centers at NYU Langone in Sunset Park are largely representative of the Sunset Park neighborhood. About 50% of Sunset Park residents are immigrants to the U.S. and over 80% speak a language other than English. Over 30% of residents live below the Federal Poverty level. Residents also represent diverse racial/ethnic groups: 36% Latinx, 4% Black, 35% Asian, and 24% White, including people of Middle Eastern descent.

The longitudinal study utilizes electronic health records (Epic) to identify potential participants who have agreed to be contacted for research purposes. Trained research coordinators check the daily schedule in the prenatal clinics and identify potential participants who meet inclusion criteria to be approached for recruitment and screening. Potential participants are then contacted by trained bilingual research staff who speak English and either Chinese (Mandarin or Cantonese) or Spanish. Staff recruit participants either in person or by phone, providing basic information about the study including a flyer describing the study and a welcome packet including a list of next steps and timing of assessments. If women express interest in hearing more, they are screened to confirm eligibility and offered the opportunity to enroll using a written or electronic informed consent form approved by the NYUGSOM IRB (S22-00242). Data collection for the longitudinal study began in May 2022 with recruitment of pregnant women in their first trimester of pregnancy. We expect to recruit 300 women a year for three years for a final sample of 900 women in the longitudinal study.

In addition, when women are consented to the longitudinal study, they are asked whether they are interested in being contacted to participate in semi-structured qualitative interviews; those women that consent will then be contacted for that portion of the study. Data collection for the qualitative component has not yet begun, but will initially involve recruitment close to the time of participants’ baseline survey. We anticipate recruiting up to 24 women per linguistic group using purposeful sampling [41] to explore themes that arise from the quantitative data collection, as well as the mother’s thoughts on early childhood development and school readiness. Future qualitative work will involve recruitment and interviews among families with older children. All interviews will be conducted in the women’s preferred language by bilingual research staff who are in linguistic and cultural affinity with participants.

*Inclusion and exclusion criteria*. In order to be eligible for the longitudinal study, women must be at least 17 years of age, have a means of contact with the study team (e.g., a working phone), be less than or equal to 18 weeks gestation, and live or receive services in Sunset Park, Brooklyn.

In addition, women will be excluded from the study if they have a known non-singleton pregnancy (e.g., twins, triplets), are unable to provide consent or do not speak English, Spanish, or Chinese, if they have a significant impairment that would prevent them from participating (e.g., schizophrenia, intellectual disability), or if they have plans to leave the neighborhood.

We expect 2–5% of women will not meet eligibility criteria, and 10–15% will not be interested in participating. Thus, we will screen approximately 1300 women in order to recruit a final sample of 900.

#### Cross-sectional study

Parents or caregivers of 2-, 4-, and 6-year-old children are currently being recruited by the same research staff at the time of well-child visits in the pediatric clinics of the Family Health Centers at NYU Langone in the Sunset Park neighborhood. The cross-sectional study also utilizes electronic health records (Epic) to identify potential participants who have agreed to be contacted for research purposes. Trained research coordinators check the daily schedule in the pediatric clinics and identify potential participants who meet inclusion criteria and should be approached for recruitment and screening. In addition, the study will be advertised at the Family Health Center Early Childhood Centers, four full-day, year-round childcare centers, and in collaboration with community organizations in Sunset Park. Parents who contact the study team will also be assessed for eligibility.

With the same expectations for eligibility and interest as in the longitudinal study (above), approximately 200 families in each age group will be screened and approached in order to obtain a final sample of approximately 150 families in each age range (150 parent-child dyads at age 2 years, 150 dyads at age 4 years, and 150 dyads at age 6 years). Data collection for the cross-sectional study began in January 2023.

*Inclusion and exclusion criteria*. In order to be eligible to participate in the cross-sectional study, parents or caregivers must be at least 17 years of age, have a child within the age range of one assessment point (2-year-old, 4-year-old, or 6-year-old), and live or receive services in Sunset Park. All exclusion criteria are the same as noted above for the longitudinal study, except that families do not need to plan to remain in the neighborhood as they are only seen one time for the assessment.

### Sample size and power

Because we will be identifying trends in family and child well-being, we do not have a specific inferential question, such as the value of a parameter or predictor that will be evaluated. Thus, no power analysis is applicable; the extent to which we identify varying trajectories of family health and wellbeing and child development is largely a feature of data variability, not sample size. However, sensitivity analysis indicates that using a repeated measures multivariate analysis of variance (MANOVA) with both within and between subjects factors, our projected final longitudinal sample of 900 will provide 80% power to detect a potential difference in long-term indicators (e.g., child development) between groups with an effect size of *d* = .17 and a Type I error rate of .05.

For the qualitative component of the longitudinal study, our final projected sample will include up to 24 participants per linguistic group, purposefully sampling mothers based on emerging themes from the quantitative data collection. We expect that we will need to interview 12 mothers per theme strata to reach saturation [42].

Using analysis of variance (ANOVA), our projected final cross-sectional sample of 450 will provide 80% power to detect a potential difference between groups with an effect size of *d* = .23 and a Type I error rate of .05.

### Variables and measures

Table 1 lists all non-outcome constructs being assessed and corresponding instruments being utilized in this study, related to sociodemographics, psychosocial stressors, health, and the home and neighborhood environment, as well as exposure to programs initiated through TGS. These instruments are being implemented through structured interviews with the prenatal women/parents/caregivers enrolled in the studies, except the Newest Vital Sign, which is a direct assessment of Health Literacy, by trained bilingual research staff who speak English and either Chinese (Mandarin or Cantonese) or Spanish.

**Table 1:** Non-outcome constructs.

Construct		Instrument	Prenatal	Age 0	Age 2	Age 4	Age 6
Sociodemographics		USDA Hunger Vital Sign (food security), [43] material hardship, housing	x		x	x	x
Psychosocial Stressors		PROMIS Depression [44]	x	x	x	x	x
		Patient Health Questionnaire-9 [45]	x				
		PROMIS Anxiety [44]	x			x	
		Pregnancy Related Anxiety Scale [46]	x				
		Everyday Discrimination Scale [47] and Perceived Immigration Policy Effects Scale (PIPES) [48]	x	x	x	x	x
		Dyadic Adjustment Scale [49]	x	x	x	x	x
		Stress (general, pregnancy related, immigration related)	x	x	x	x	x
		Family Problem Solving [50]	x		x	x	x
		PRAMS domestic violence [51]	x				
Prenatal Health & Care	PRAMS Prenatal physical health [51]		x				
	PRAMS Care Utilization [51]		x				
	Discrimination in Medical Settings [52]		x				
	Care satisfaction		x				
	PROMIS Social Support [53]		x				
	Health Literacy (Newest Vital Sign) [54]		x				
Home & Neighborhood	Plans for child care		x				
	Cultural practices		x			x	
	Neighborhood Cohesion [55]		x			x	
Program participation/utilization, including TGS/NYULB programs			x	x	x	x	x

Table 2 lists all outcome constructs being assessed in this study, including items related to both the parent (parent-child interaction, stress) and the child (language development, self-regulation). These constructs are being assessed through interviews, observations, and direct assessments of the child’s abilities by trained bilingual research staff who speak English and either Chinese (Mandarin or Cantonese) or Spanish.

**Table 2:** Outcome constructs.

Construct	Instrument	Type of Assessment	Age 0	Age 2	Age 4	Age 6
Child Measures							
Child Development/ Milestones	Ages & Stages Questionnaire (ASQ) [56]	Interview		x	x	x
Child Language Development	Naturalistic assessment of child language	Observation		x	x	
	Peabody Picture Vocabulary Test (PPVT) [57]	Direct Assessment			x	x
	Receptive One-Word Picture Vocabulary Test (ROWPVT) [58]	Direct Assessment			x	x
	Language exposure (home, child care, early child education)	Interview	x		x	x
	MacArthur-Bates Communicative Development Inventory (CDI) short form [59]	Interview		x		
Child Self-Regulation	Laboratory Temperament Assessment Battery (Lab-TAB) [60]	Observation	x			
Child Executive Function/Self-Regulation	Dimensional Change Card Sort Test [61]; Head Toes Knees Shoulders Task [62]	Direct Assessment			x	x
Child Behavior	Behavior Assessment System for Children subscales (BASC) [63]	Interview			x	x
Child School Readiness	Bracken School Readiness Assessment [64]	Direct Assessment			x	x
Parent-Child & Environment Measures							
Parent-Child Interaction	Adult-Child Interactive Reading Inventory (ACIRI) [65]	Observation			x	
	semi-structured interactions coded using Parent Child Interaction Rating Scale-Infant Adaptation (PCIRS-IA) [66]	Observation	x	x	x	x
Home Environment & Family	Childcare: Plans, Early Childhood Education (ECE), Early Intervention (EI)	Interview	x	x	x	x
	Cultural practices	Interview			x	
	StimQ Cognitive Home Environment [67]	Interview	x	x	x	x
	Chaos Hubbub And Order Scale (CHAOS) [68]	Interview	x	x	x	x
Parent Measures							
Parenting	PRAMS feeding [51]	Interview	x		x	x
	Routines: sleep, screen time	Interview		x	x	x
	Parent Engagement of Families from Latino Backgrounds (PEFL) Questionnaire [69]	Interview	x	x	x	x
	Parenting Self-Agency Measure [70]	Interview	x		x	
	Parent Reading Beliefs Inventory [71]	Interview	x		x	
	Parenting Daily Hassles [72]	Interview				

The constructs and associated instruments were chosen based on lessons learned from previous formative research conducted by TGS and C3 investigators; the most culturally-relevant instruments were selected. Measures that have not been previously validated in Spanish or Chinese were reviewed by study team members who share the cultural and linguistic backgrounds of the Sunset Park participants to assess for both translation and cultural relevance to ensure validity for the target populations. Nonetheless, given that most of these measures were developed in white communities, we use them cautiously in this population, and are pairing our quantitative surveys and assessments with qualitative interviews that will be implemented with a subset of the participants.

### Planned analyses

#### Longitudinal study

First, we will score all validated scales according to manual guidance or precedent set by the most updated published validation papers. Then, factor analysis using principal axis factoring for extraction of data and rotation (e.g., varimax) will be used to identify and confirm underlying constructs in the larger set of variables in order to reduce collinearity. This will be particularly important in the scales that have not previously been validated in Chinese and Latinx communities. Descriptive statistics will be used to characterize the distribution of baseline variables, including means, standard deviations, and frequencies.

Longitudinal analyses will then be conducted to examine trajectories of family and child outcomes over time in accordance with appropriate statistical methods for observational data [73]. These analyses will include both linear mixed models, which allow for the addition of random effects to account for the non-independent nature of repeated measures, and latent-class mixed models, which allow for the identification of subpopulations within the sample based on differing trajectories. Structural equation modeling will also be used to examine pathways through which TGS program participation is associated with outcomes including the assessment of both direct and indirect effects along with potential subgroup trajectories. We will use the Comparative Fit Index (CFI) and root mean square error of approximation (RMSEA) to determine model fit and will test the significance of direct and indirect effects of TGS program participation and other sociodemographic predictors (e.g., income/material hardship, education) on the short- (family health and wellbeing) and long-term (child development) outcomes of the C3 Study. All analyses will be adjusted for potential confounders identified through descriptive analysis.

For the qualitative component, once transcribed in the language of interview and translated into English, the interview data will be managed and analyzed using qualitative software. Following a process of data familiarization (i.e. reading and re-reading of the interviews) the transcripts will be independently coded by at least two bilingual team members. Analyses will be carried out following the thematic analysis guidelines provided by Braun and Clarke [74]: (1) familiarizing oneself with the data; (2) generating codes; (3) constructing themes; (4) reviewing potential themes; (5) defining and naming themes; and (6) writing up the findings. The qualitative research team will meet regularly during all phases of the study to discuss code book development, the coding process, and interpretation, ultimately enhancing the confirmability of the research findings [75].

All quantitative findings will be interpreted jointly with the qualitative findings for maximum relevance and understanding.

#### Cross-sectional study

As above, we will score validated measures and use factor analysis to identify underlying constructs. Factor analyses utilizing principal axis factors will be used to identify constructs in the larger set of variables and reduce collinearity. Descriptive statistics will first be examined to characterize the distribution of the variables, including means, standard deviations, and frequencies, and appropriate analyses will be selected based on the normality or non-normality of the data. We will examine differences between groups in short- and long-term C3 Study outcomes based on family and child characteristics, birth order, family-level risks (e.g., maternal depression, anxiety), and sociodemographic variables (e.g., income/material hardship, education) using analysis of variance (ANOVA/MANOVA), Kruskal-Wallis ANOVA for non-parametric data, and other multivariate methods appropriate for cross-sectional analysis [73].

#### Missing data

Multiple imputation techniques, including Full Information Maximum Likelihood estimation [FIML; 76] will be used to recover item-level missing data using the other concurrently collected variables. Item-level missing data is expected to be minimal as the research team has expertise in minimizing missing data. However, we will consider fully imputed models as a sensitivity check to ensure that the original target population is well-represented in both the longitudinal and cross-sectional analyses.

### Ethical considerations

The safety and comfort of participants is a priority in this study. As such, all study procedures, informed consent forms, and recruitment materials have been approved by the NYUGSOM IRB (S22-00242). At the time of recruitment, participants are given detailed information about the study verbally and through a written informed consent form and briefer “key information” form in the participant’s native language. During the consent process, trained study personnel read the informed consent form with the participant in detail, including the nature of the study, inclusion and exclusion criteria, a description of each assessment, and all risks and benefits. The contact numbers of senior study staff are listed on the consent form and will be pointed out to participants during this time. Participants are informed that participation is voluntary, that their decision to participate or not will not affect the care they receive, and that they can withdraw their enrollment at any time. Signed consent is obtained after all participant questions have been answered and it is clear that they have an understanding of the study procedures.

The main ethical consideration in this observational study is participant and data confidentiality. Participant contact information is securely stored electronically on password protected networks behind NYU institutional firewalls and will not be connected to study data. Instead, participants are assigned a unique study identifier (ID) which will be used for all assessments and data processing and analysis. Only approved study personnel have access to the database linking study IDs with participant contact information. All assessment data, including questionnaires, video-recordings, and other measure data, are kept in locked file cabinets in locked rooms, or electronically with password protection and appropriate firewalls and encryption. Moreover, we have obtained a Certificate of Confidentiality (CoC) from NIH to protect the privacy of subjects in this research study. CoCs prohibit disclosure of identifiable, sensitive research information to anyone not connected to the research except when the participant consents or in a few other specific situations.

Additional ethical considerations include discomfort of adult or child participants due to embarrassment or discomfort in disclosing private information during interviews or in being observed during interactions with their child. In order to address this risk, participants are reminded that all portions of the assessments are voluntary and that they can skip any questions or portions with which they do not feel comfortable. Study personnel are trained to identify indicators of discomfort in both adults and children, and offer to answer questions, remind participants of the confidentiality of their information, and suggest skipping portions, taking a break, or discontinuing the assessment as appropriate. In addition, if participants endorse any self-harm or harm to others, we have an emergency protocol in place to immediately contact a licensed psychiatrist for assistance.

Lastly, the C3 Study is community-engaged and community-driven at every stage. The TGS team has worked with community stakeholders in Sunset Park to develop research questions, interpret study findings, and identify initiative priorities, and the C3 study is leveraging those connections to develop research priorities, as well as the ongoing TGS relationships to foster community engagement. The study team will continue to meet with community members to clarify community attitudes toward study design and evaluation, discuss strategies for recruitment and engagement, review findings and ways to present results, as well as dissemination to key stakeholders.

### Dissemination

Given the centrality of community in the C3 Study aims and design, first and foremost, our dissemination efforts will include close discussion and collaboration with the community. This includes the prenatal and pediatric clinics from which we are recruiting in terms of broader trends in the findings, as well as the families themselves if we detect any positive (atypical) surveillance, screening, or evaluation results. We will regularly share updates with the NYU Langone Family Health Center Research Steering Committee, and seek out additional venues for feedback from the community on the interpretation of findings. For example, we have already held one such meeting at the Sunset Park Early Childhood Research Collaborative, a forum for researchers and practitioners collaborating in Sunset Park to come together on a bi-monthly basis to discuss relevant work being done in the community. Such work includes prenatal education and counseling, systems-level work to address childhood early adversity, and our own C3 Study. During this meeting we shared aggregated, de-identified preliminary baseline survey results (so there was no risk of identification of individual participants) and solicited feedback from practitioners and clinicians in Sunset Park to help put our initial results into context. Some of the feedback that emerged from this meeting is helping to form the basis of our interview protocol for the qualitative component of the C3 Study.

In addition to our community partners, there is a tremendous interest in academia in research on place-based initiatives, and the findings from the C3 Study will appeal to a diverse academic audience from many fields including pediatrics, public health, education, psychology, epidemiology, and others with policy implications on how to improve quality of life and access to opportunity. We will present our work at a variety of national conferences and publish research findings in peer-reviewed journals using the STrengthening the Reporting of OBservational studies in Epidemiology (STROBE) checklist in order to provide complete and accurate reporting of the study. In line with the goals of our study, we will make the findings accessible to the broader public in other formats, including a public use dataset with deidentified data that can be shared to interested parties.

## Conclusions

The C3 Study will aid in our knowledge and understanding of the assets and vulnerabilities of families in Sunset Park, Brooklyn, and the role TGS can play as a community initiative in strengthening the prenatal through school entry periods. It will have a particular emphasis on family engagement in different programs and the potential synergistic benefits of focusing on individuals, communities, and systems by centering interactions between individuals and their broader environmental contexts. Lastly, it will further add to the knowledge base about the efficacy of place-based initiatives that seek to improve quality of life and invigorate urban renewal.
